# Understanding the Odour Spaces: A Step towards Solving Olfactory Stimulus-Percept Problem

**DOI:** 10.1371/journal.pone.0141263

**Published:** 2015-10-20

**Authors:** Ritesh Kumar, Rishemjit Kaur, Benjamin Auffarth, Amol P. Bhondekar

**Affiliations:** 1 CSIR-Central Scientific Instruments Organisation, Chandigarh, India; 2 Academy of Scientific and Innovative Research, New Delhi, India; 3 Nagoya University, Nagoya, Japan; 4 Neuroinformatik, Department of Neurobiology, Freie Universität Berlin, Berlin, Germany; MRC-National Institute for Medical Research, UNITED KINGDOM

## Abstract

Odours are highly complex, relying on hundreds of receptors, and people are known to disagree in their linguistic descriptions of smells. It is partly due to these facts that, it is very hard to map the domain of odour molecules or their structure to that of perceptual representations, a problem that has been referred to as the Structure-Odour-Relationship. We collected a number of diverse open domain databases of odour molecules having unorganised perceptual descriptors, and developed a graphical method to find the similarity between perceptual descriptors; which is intuitive and can be used to identify perceptual classes. We then separately projected the physico-chemical and perceptual features of these molecules in a non-linear dimension and clustered the similar molecules. We found a significant overlap between the spatial positioning of the clustered molecules in the physico-chemical and perceptual spaces. We also developed a statistical method of predicting the perceptual qualities of a novel molecule using its physico-chemical properties with high receiver operating characteristics(ROC).

## Introduction

There are not many answers to the question of why a molecule smells as it smells. Researchers have been working to find models that can predict how a molecule smells based on its physico-chemical properties [1,2]. The first hurdle itself has been hard to fathom i.e. how do you objectively define perceptual descriptors? At first, researchers tried to tackle this problem by defining primary or basic descriptors much like in vision and audition [3], but the conclusions never converged to a well-defined result. Similarly, efforts towards defining specific molecular properties which could account for a very specific perceptual descriptor (e.g. "musk") were undertaken [4–6]. However, these also failed to define a general rule to predict the perceptual descriptor of a molecule.

Research has also gravitated towards defining the perceptual classes, or in general the perceptual primaries (basic categorical dimensions or the number of dimensions explaining the olfactory perceptual descriptors), based on various databases and literature using statistical techniques. The most important and recent works are by Mamulok *et al*. [7], Castro *et al*. [8], Kulakouv *et al*. [9] and Zarzo *et al*. [10]. Although, the research on defining olfactory primaries have been a significant step, the methods still need to be intuitive and tested on larger databases [11]. For example, Mamulok *et al*. [7] used the concept of sub-dimensional distance along with multidimensional scaling (MDS) and self organizing maps (SOM) having a toroidal architecture, the interpretation of which becomes difficult due to the lack of explanation regarding the physical significance of the model. The use of non-negative matrix factorization (NMF) by Castro *et al*. [11] has been tested only on the Dravenieks database, thereby limiting the generality of the model. Moreover, the NMF method itself has instability issues and the simulations have to be repeated to get a common result [12], though statistical methods have been used to avoid instability. Further, the linear combinations of the actual perceptual descriptors also causes a significant interpretation issue. Zarzo *et al*. [10] have used principal component analysis on many perfumery databases and defined perceptual groups. Although a significant step, this type of analysis suffers from two issues, firstly the perceptual databases are known to be very sparse and hence the interpretation of linear combination of perceptual descriptors becomes very difficult, secondly the distance measure in this case can be very misleading [13]. The number and dimension of distinct smells which can be discriminated by humans is still an open question, which has more recently been very aptly put forward by Gerkin and Castro [14] and Meister [15].

The present work is an attempt to at first gather as many diverse datasets as possible, i.e. perfumery, food and agriculture, drug etc. [16–20] and then give a visual representation to perceptual universe, wherein even a layman can see the perceptual similarities, understand the groups and easily interpret the results. To this end, we have accumulated publically available odour databases consisting in total 526 perceptual descriptors and 3016 molecules. We then present a network based approach to explore the perceptual space and investigate the underlying similarity of perceptual descriptors and define perceptual communities across all databases. The odour networks follow a power law and are significantly different from random networks meaning the presence of preferential attachment and presence of hubs. It may mean the dominance of association of major perceptual descriptors and suggest towards the vagueness of our vocabulary or a local clustering. We have also explored an important question that, is the positioning of perceptual descriptors in the odour network merely due to the semantic relatedness of the words, by comparing it with a widely used semantic database (Brown database) using a bag of words approach. The odour networks show a marked variation from the semantic networks indicating that, the positioning of the descriptors is not only caused by the semantic-relatedness of the words. The user can visualize these networks at http://odornetwork.com/network/index.html.

One of the most striking results coming out of the efforts on finding structure-odour-relationship is the representation of physico-chemical properties in a low dimensional space whose principal axis correlates with the “pleasantness” [10,21–28]. With the advent of software providing a large number of physico-chemical properties and better understanding of olfactory mechanisms, there has been some important works towards development of pleasentness prediction models of molecules [29] and their correltions with neural responses [21]. The research on finding a systematic relationship between physico-chemical properties of molecules and perceptual descriptors has been significant, yet the works are concentrated on smaller databases (e.g. by Khan *et al*. [29]) for the pleasantness prediction. Schmuker *et al*. [30] have predicted perceptual qualities based on the Sigma-Aldrich database, but their main focus was on designing a virtual receptor and to demonstrate its significance. Our work in this regards is significant from two perspectives. First, we accumulate and curate a diverse open domain dataset of 3016 molecules along with their 1666 physico-chemical properties (effectively 1489 properties after pre-processing) as mentioned above. Second, we show that the molecules if projected to separate non-linear space in perceptual and physico-chemical dimensions, they respectively occupy similar positions. Hence, a model can be developed which can predict the perceptual qualities of the molecules just by using the physico-chemical properties. This, we have validated by designing a random forest classifier and obtained very good ROC values. We have successfully extracted some useful features for all the databases separately and combined. Using this framework we can directly predict the perceptual qualities of a novel molecule using its physico-chemical feature.

## Results

### Statistical analysis of perceptual data

**Fig 1:**
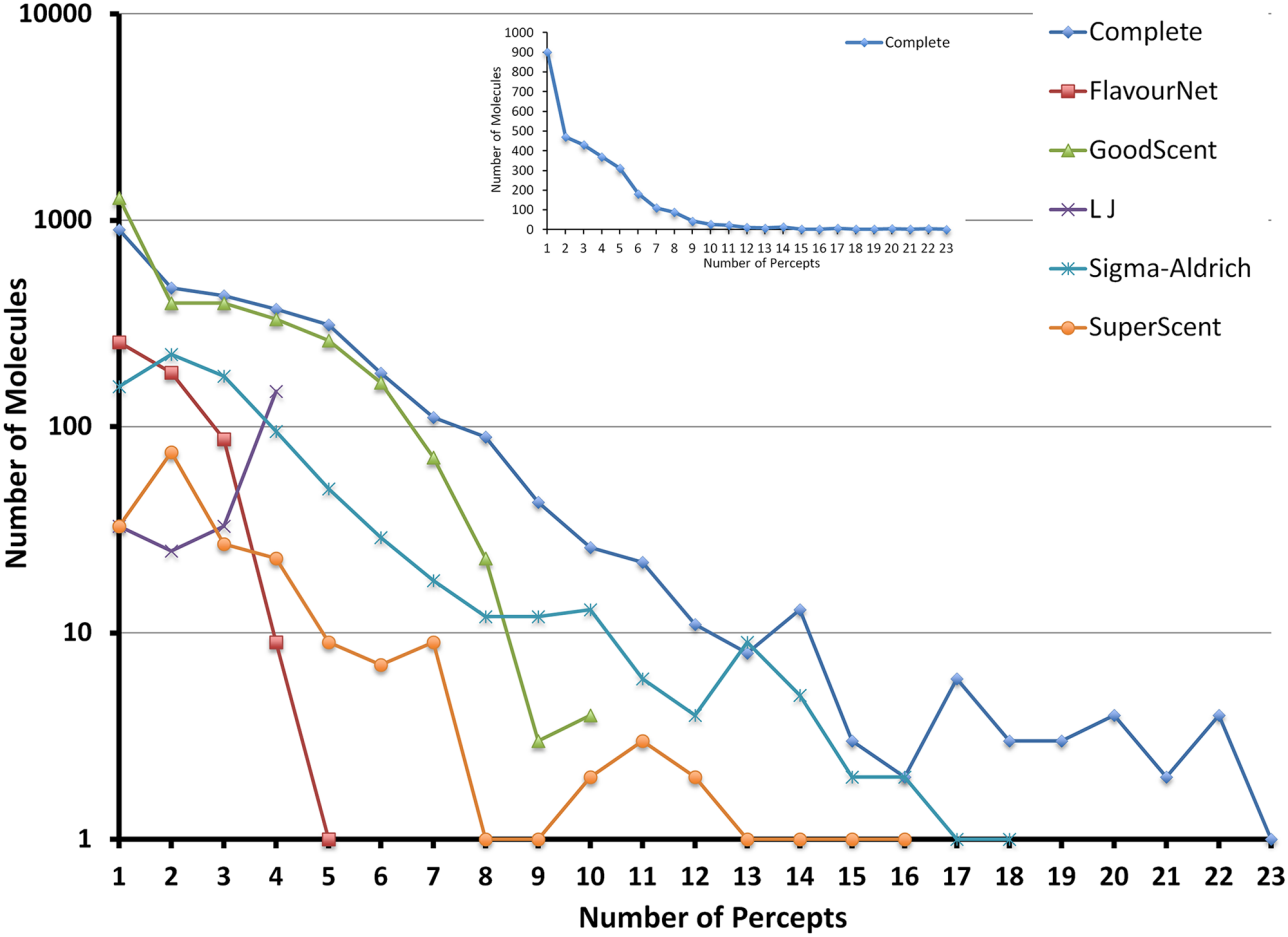
Database characteristics: The number of perceptual descriptors vs number of molecules per database.

At first, we analyse database characteristics and their underlying perceptual dimensions. Table 1 shows the number of molecules, number of perceptual descriptors, average number of perceptual descriptors per molecule (*AP*_*m*_), average occurrence of a perceptual descriptor (*AM*_*p*_), and percentage of sparseness (*S*_*p*_ defined in methods section) in the different databases. It can be observed that on an average a molecule has been described by a very few number of perceptual descriptors and very few molecules have been described by larger number of perceptual descriptors (see Fig 1, the inset figure depicts the y-axis of complete database in linear scale). This trend however is different in Leon and Johnson database (LJ) where most of the molecules have more than three perceptual descriptors. A look at the sparseness data indicates all the databases are very sparse with GoodScents database being the sparsest and SuperScent database to be the least sparse. Further delving into the description shows the dominance of association of some perceptual descriptors like ‘fruit’, ‘sweet’, ‘floral’ along with ‘sulphur’ and ‘pungent’ etc. in all the databases (see S1 Table for top ten occurring perceptual descriptors). The databases may be partitioned into a small subset of words that are associated with a large number of molecules, thereby suggesting the possibility of creating odour classes. Also, a smaller group of words associated with a relatively larger number of molecules may indicate specificity in the odour representation. It has also been observed that the word frequencies (see S1 Table top ten occurring words) were almost the same across all the databases which reveal a common process of classification.

**Table 1:** Database Characteristics.

Dataset	No of molecules	No of perceptual descriptors	Avg No of perceptual descriptors per molecule	Avg occurrence of a perceptual descriptor	Sparseness (%)
**Flavornet**	537	177	1.72 (1–5)	5.226 (1–58)	99.03
**GoodScents**	2933	456	2.60(1–10)	16.72(1–689)	99.43
**LJ**	239	157	3.23 (1–4)	4.929(1–97)	97.94
**Sigma-Aldrich**	815	107	3.44 (1–21)	26.20(1–196)	96.78
**SuperScent**	196	95	3.48(1–19)	7.18 (1–80)	96.33
**Complete database**	3017	526	3.51(1–23)	20.11(1–830)	99.33

The values in brackets represent the range of corresponding column descriptors

### Co-occurrence network of perceptual space

We can describe each database as an undirected graph or network, where nodes are perceptual descriptors and an edge is shared by two perceptual descriptors if they have occurred together in the perceptual description of a molecule. It should be noted that the perceptual descriptors thus forming the nodes may have multiple edges between themselves. We look at these networks separately for each database.

The important questions to be addressed with respect to the perceptual network are about its structural organisation, particularly, its difference from a random network and its degree distribution. We also sought to understand whether the positioning of the perceptual descriptors is only due to their semantic relatedness.

In general, random network models play an important role in standard network analysis as they serve as null templates against which the non-randomness of the networks could be tested [31]. A random network follows a Poisson degree distribution, a special case of Gaussian distribution. The Poisson and Power distributions differ radically. The main feature of the Poisson distribution can be entirely characterized by its mean and variance [32]. A Power distribution on the other hand does not have a well-behaved mean or variance. Hence, no mean and finite standard deviations can be assumed to be present for a power law which can be used to represent the typical features of the distribution and to base confidence intervals [33]. Power law seems to be ubiquitous, they have been found to be both in natural [34] and man-made systems internet [35], cities ranked by population [36] etc.

**Fig 2:**
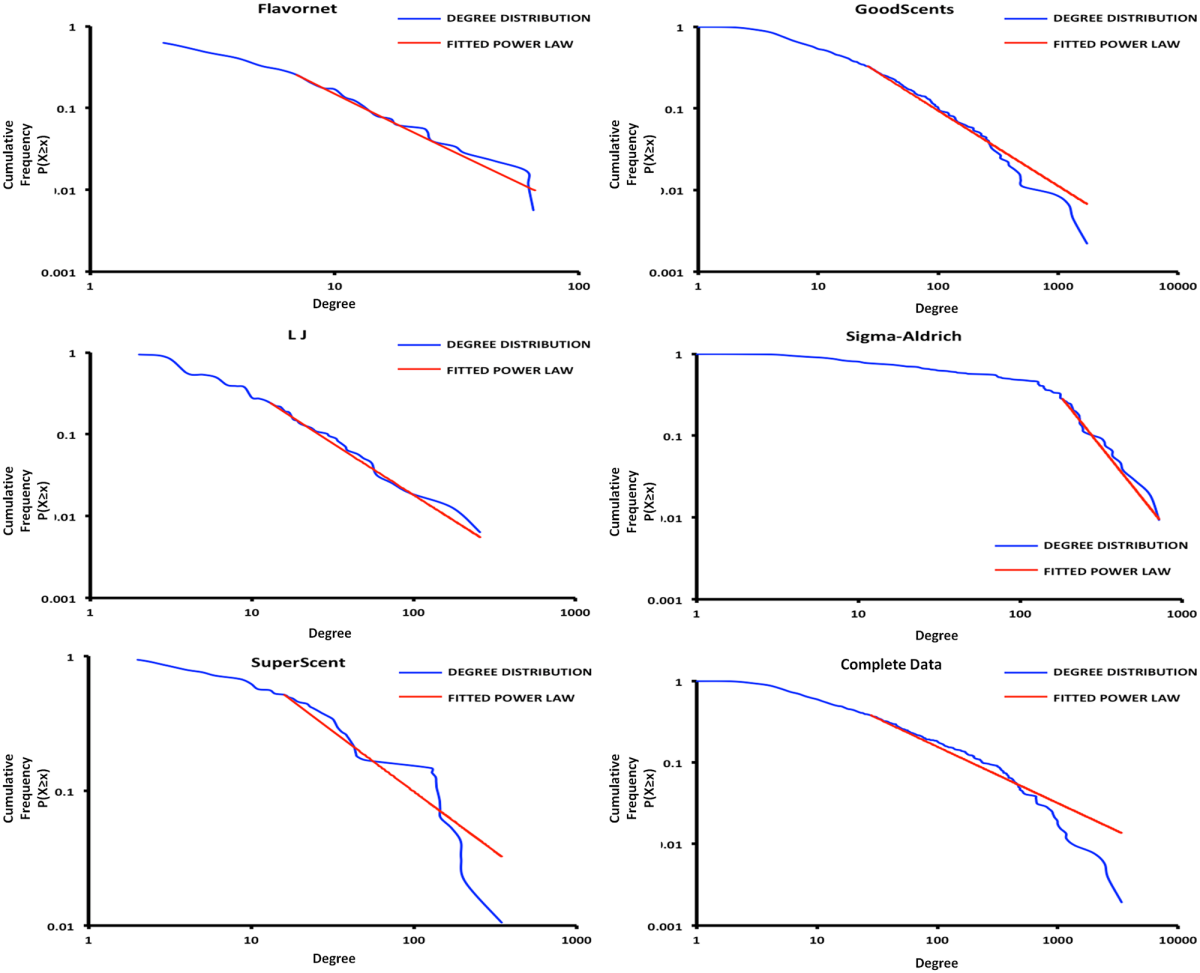
Degree Distribution. The degree distribution of the networks in log-log plot along with the fitted truncated power law. For all the networks, except SuperScent, the probability that a given perceptual descriptor connects with k other perceptual descriptors follows a power law (p(x) = x^-α^) with α Є [2, 3] or α ~ 2.

For each database, a corresponding random network having same number of edges and nodes (as the perceptual network) was generated using Erdos-Renyi G(n,m) model [37] (see S1 Text for details). 1000 such instances of these random networks were created and their clustering coefficients were calculated (see Table 2). Clustering coefficient quantifies the extent to which the neighbours of the concerned node are connected to each other. It can be used to differentiate a scale free network from a random network (see details in S1 Text). The clustering coefficient distributions were compared with the clustering coefficient values of the corresponding odour networks. Significant difference was observed using z-test (p-values < 0.001), indicating that the null hypothesis (i.e. the clustering coefficient of the databases are similar to the random network) can be rejected. Further, to understand the topology of the networks, the degree distributions of the perceptual descriptors were analysed. For all the networks, except SuperScent, the probability that a given perceptual descriptor connects with k other perceptual descriptors follows a power law (p(x) = x^-α^) with α Є [2, 3] or α ~ 2 [38]. Fig 2 shows the degree distribution of the networks in log-log plot. For all the databases the X_min_ value happens to be at a reasonably smaller value except Sigma-Aldrich. This may be attributed to the larger graph density (0.329) for Sigma-Aldrich in comparison to the other databases. Specifically, there are more number of connections between nodes of Sigma-Aldrich which indicates that, even the sparsely represented perceptual descriptors on an average are connected to more number of perceptual descriptors than the other databases. This may indicate better curation of the Sigma-Aldrich database and perhaps more representative of perceptual descriptors.

**Table 2:** Network Characteristics of the odour network and the comparison with random network where, A = Avg degree, N_d_ = Network diameter, N_l_ = Avg path length, D_g_ = Graph density, α = Power law exponent, X_min_ = Power law cutoff degree, r = Assortativity Coefficient, cl^avg^ = Clustering Coefficient and *R-cl* ^*avg*^ = Random Clustering Coefficient.

Database	#Nodes	#Weighted Edges	A	N_d_	N_l_	D_g_	α	X_min_	r	cl^avg^	*R-cl* ^*avg*^
**Flavornet**	177	508	5.74	6	2.98	0.024	2.41	7	-0.13	0.34	*0*.*0223 ± 0*.*0069*
**GoodScents**	456	11057	48.50	5	2.33	0.041	1.92	26	-0.17	0.74	*0*.*0412 ± 0*.*0013*
**LJ**	157	1012	12.90	6	2.49	0.05	2.25	13	-0.18	0.73	*0*.*0498 ± 0*.*0067*
**Sigma-Aldrich**	107	6655	124.40	4	1.73	0.329	3.46	183	-0.14	0.84	*0*.*3296 ± 0*.*0039*
**SuperScent**	95	1850	38.95	4	2.07	0.167	1.89	16	-0.18	0.73	*0*.*1673 ± 0*.*0072*
**Complete database**	526	25805	98.12	4	2.21	0.054	1.69	28	-0.20	0.80	*0*.*0542 ± 0*.*0012*

The power law distribution also gives rise to a phenomenon of being “scale free”[32]. It indicates that the olfactory space is dominated by a few most frequently occurring perceptual descriptors or hubs in a network, thereby suggesting preferential attachment of the perceptual descriptors. The dominance of these hubs in almost all the networks suggests universality of the olfactory perception. We listed the top ten hubs based on the degree of nodes for each network resulting in total of 60 hubs. Then, we observed the number of times each hub has appeared across all networks. e.g. "fruit", "floral", "wood "and "herb" were among the top ten hubs in all networks and "sweet", "green" were hubs in all the networks except SuperScent. Table 3 shows these hubs according to the number of times they have occurred in the networks in decreasing order (6, 5, 3, 2, 1 times). Taking a closer look at the nodes having higher degree reveals the inability of the subjects in verbally describing the odours. A similar kind of observation has been reported in linguistic research in which the frequency of occurrence of a particular word has been seen to be inversely related to the rank of that word in the corpora [39]. The underlying origin of such phenomenon has been given the name as "theory of least action", i.e. people tend to speak those words which they think will convey the broadest of information on a given topic, which is true for expert or a layman [40,41]. So, in the event of smell reporting, the subject would tend to speak first the broader meaning words and then she/he would tend to speak more specific and related words. Moreover, odour perception in general is associated with objects which we encounter and it is an integration of inherent odour characteristics with the psychophysical condition of a perceiver. Odour association with objects becomes difficult for a perceiver in absence of a visual cue [42].

**Table 3:** Hubs in the Network.

Number of occurrences	Perceptual descriptors
6	Fruit, Floral, Wood, Herb
5	Sweet, Fat, Green
3	Nut, Citrus
2	Pungent, Meat, vegetable
1	Balsam, Sulfur, Wax, Earth, Ether, Pineapple, Spice, Apple, Chocolate

Further, we calculated the assortative index [43] of all perceptual networks (see Table 2). The assortative index quantifies the property of preferential attachment amongst nodes. It can be defined as the Pearson correlation coefficient of degree between pairs of linked nodes (S1 Text). The negative values of the indices indicate that the high degree nodes preferentially connect with low degree nodes and vice versa. The negative values obtained for all the databases (see Table 2) suggest that the perceptual descriptors with broader meanings are connected with very specific descriptors and, these perceptual descriptors can be merged together for classification purposes. This is a typical characteristic of an unconstrained evolutionary network having tendency to evolve towards its maximum entropy state [43,44]. This characteristic of network makes it disassortative. This disassortativeness could be exploited for the identification or creation of perceptual classes.

In order to understand whether the odour network presented here are just capturing the semantic relatedness of the words, we identified a general text corpus, brown corpus consisting of different text categories [45]. We further generated a semantic co-occurrence network out of this corpus (details in method section). Table 4 describes the general characteristics of the 6 networks generated. It should be noted that, we searched for only those words which were in our olfactory perceptual descriptor list. Obviously, we were not able to find all the words from our databases. For a fair comparison, we chose only those words which were found in the corpus and extracted the subnetworks of the corresponding olfactory databases. It can be very easily observed from the Table 4, that the average degree and graph density of the semantic network is lesser. Whereas, the network diameter and average path length are longer than that of the corresponding odour networks. This points towards a very obvious proposition that the semantic network is far more sparse, indicating that people generally do not use the olfactory terminologies together in their writing or speaking. Before moving on to the objective comparison between these networks, it is worth noting that the clustering coefficient of the semantic networks are far lesser than their counterpart odour networks, indicating very low local clustering affirming the argument made earlier.

**Table 4:** Semantic analysis and comparison of the odour network using the brown database. The networks have been created using a bag of words approach using window sizes of 2,3,4 according to the average number of perceptual descriptors per molecule in each database. The odour subnetworks consisted of only those perceptual descriptors that were found in the semantic database. Network Characteristics along with Eigen value similarity of the perceptual network in comparison with random network has been tabulated, where, N = Number of nodes, WE = Number of Weighted Edges, A = Avg degree, N_d_ = Network diameter, N_l_ = Avg path length, D_g_ = Graph density, r = Assortativity Coefficient and cl^avg^ = Clustering Coefficient

		Semantic network							Odor Subnetwork							Eigen Value similairty
Database	N	WE	A	N_d_	N_l_	D_g_	r	cl^avg^	WE	A	N_d_	N_l_	D_g_	r	cl^avg^	
**Flavornet**	98	70	1.43	10	4.21	0.009	0.272	0.013	240	4.89	6	2.89	0.037	-0.158	0.219	3.42e+03
**GoodScents**	204	389	3.81	12	4.71	0.007	0.188	0.03	5006	49.07	5	2.21	0.078	-0.168	0.674	3.12e+06
**LJ**	80	118	2.95	7	3.55	0.015	0.189	0.018	400	10	7	2.49	0.079	-0.166	0.527	1.39e+04
**Sigma-Aldrich**	75	76	2.03	9	4.02	0.013	0.189	0	4388	117.01	3	1.57	0.429	-0.116	0.811	1.50e+06
**SuperScent**	72	73	2.03	12	5.24	0.014	0.079	0	1350	37.50	4	2.01	0.201	-0.167	0.736	2.77e+05
**Complete database**	221	218	1.97	12	4.64	0.007	0.125	0.035	13125	118.78	5	2.08	0.12	-0.210	0.764	1.80e+07

The assortativity index in the semantic networks are also positive as opposed to odour networks. This indicates that the higher degree nodes are in general connected to higher degree nodes only. This means that the broader meaning words are connected together and hence, merging of specific words to the broader meaning words for defining classes can be difficult. Table 4 lists the eigen similarities (calculation method described in methods section) of the semantic networks and the corresponding odour networks. The eigen similarity gives an unbounded measure and a value close to 0 means higher similarity. The higher eigen similarity values here indicate very low similarity between the networks.

Besides having mathematical measures, it is imperative to look into the connections between the words in the networks. We sought to understand it in the combined semantic network and the odour subnetworks. Here, some interesting observations come to fore, e.g. "fruit", in the odour network is the most commonly used descriptor which usually co-occurs with, "green", "apple", "herb" etc.; whereas, in the semantic network descriptors like, "apple", "pineapple", "banana", "vegetable", "cereal", "pepper" etc. co-occur with it. This may be due to the manifestation of food habits and culinary practices on semantics. Similarly, the descriptor "wax", in the odour network co-occurs with "fat", "green", "oil", "citrus", "cream" etc., while it co-occurs with "oil" and "paper" in semantic networks, suggesting overlaps between semantic relatedness and olfactory sensation. On the contrary, the descriptor "fish" co-occurs with "sulfur", "onion", "nut", "roast" in the odour network, and "vegetable", "soup", "skin" etc. in the semantic networks indicating that it is not the semantic relatedness which always determines the co-occurrence of the perceptual descriptors in the odour network. One can have a look at this semantic network at http://www.odornetwork.com/ under visualization semantic networks tab. The perceptual descriptors found from brown database in all databases has been enumerated in S3 Table.

### Community detection in perceptual network

**Fig 3:**
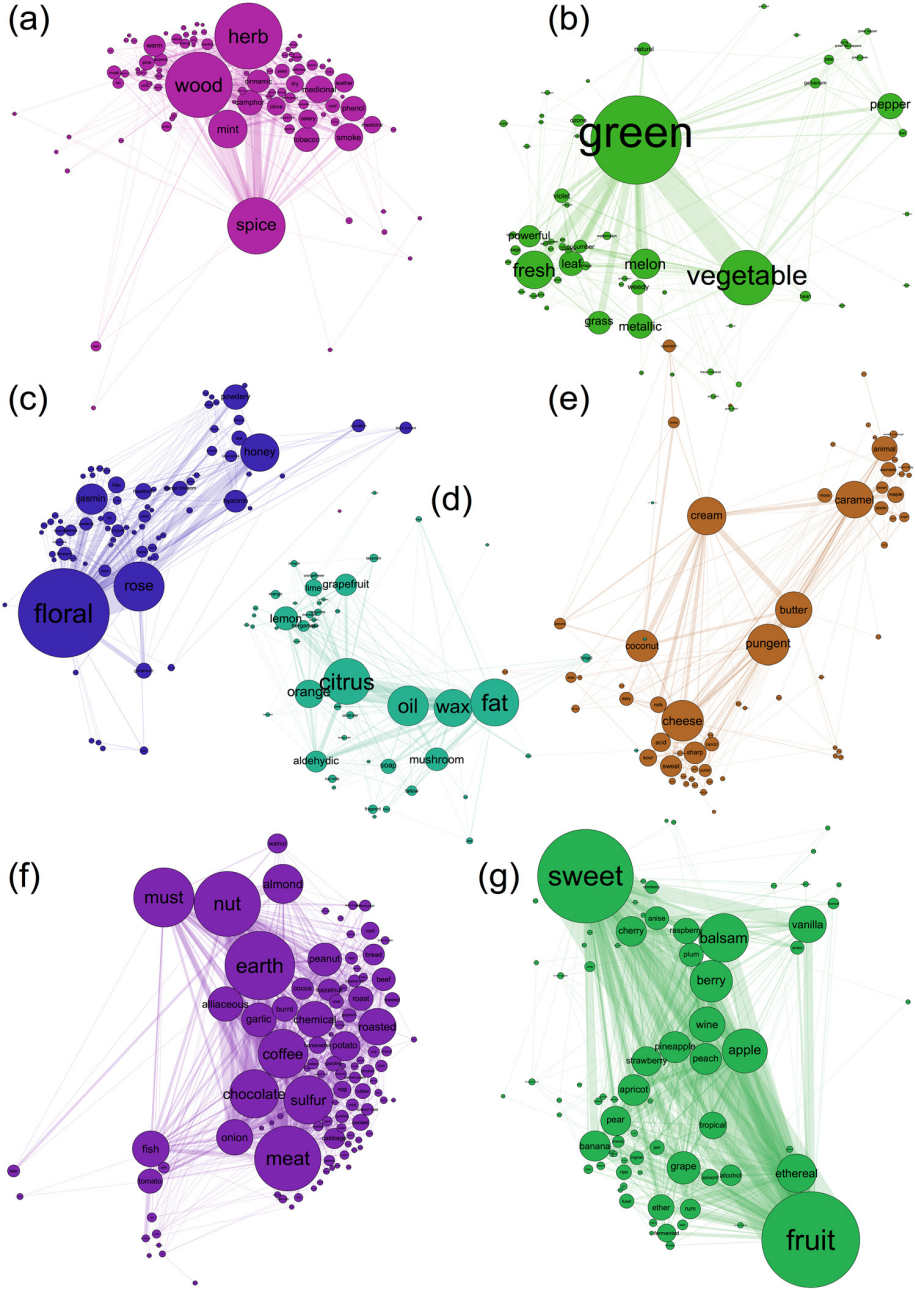
Odour Network. **(a-g)** The communities detected in the odour network of databases using modularity maximization algorithm. The colours indicate the different communities.

The study of community detection in the networks involves partitioning the graph into communities based on some objective function, where intra-community connections are dense and inter-community connections are very sparse. Here, we have used the modularity maximization algorithm proposed by Blondel *et al*. [46] (see S1 Text for details). We found 7 major communities in the combined perceptual co-occurrence network using modularity maximization. These communities individually represent a certain class of perceptual quality which can be further refined using same method. As it can be observed in Fig 3, one of the perceptual groups is of “cream”, “butter”, “cheese”, “caramel”, “animal” etc. The basis of these perceptual descriptors coming together can be attributed to the similarity of perception and sometimes source. Similar observations have been reported by others [10,47,48] (e.g. the notes [“medicinal”, “mint”, “smoke” and “phenol”]^10^, [“smoke” and “leather” and “mint”, “camphor” and “pine”]^32^), and these facts can also be observed in our case, where all these perceptual descriptors are grouped together in the same community. The descriptor “fatty”, is generally used to describe smells that relate to oil or wax, “fatty” along with “tallowy”, “aldehydic” and “soapy” are produced by short-chained aliphatic aldehydes and hence can be regarded as belonging to the same group. This fact was also observed by Muller [48]. The community of “fish”, “onion”, “sulfur”, “garlic”, “meat”, “alliceous” along with “earth”, “burnt, “coffee”, “roast”, and “bread” suggests the culinary part of the perceptual space. The databases collected here are a mix of volatile molecules from perfumery, food and agriculture, flavour and fragrance and human odour space. Hence, we see the emergence of this kind of perceptual class. We believe this part of human odour space is not biased by perfumers’ observations, but generalized perceptions. These smells if presented individually, will usually elicit unpleasant notions from the subjects, but, when presented with cues e.g. food with garlicky smell will usually result in a pleasant response. Similarly, for “onion”, “meat”, “beef” etc., these smells are learnt along with the other objects and are mostly part of our cooked food items. A visual representation can be seen at http://odornetwork.com/network/index.html. A user can see all the network properties, the groupings and the connections for the combined database here. The communities data can also be seen in the S4 Table.

### Perceptual vs Physico-chemical

The molecules in all the datasets were clustered based on their perceptual descriptors and physico-chemical properties separately. For this, spectral clustering using X-means with BIC values (criteria for getting the statistically optimal number of clusters, see S1 Text) was performed. The co-occurrence measure was used for calculating the perceptual similarity matrix, whereas the locally scaled Euclidean distance was used for calculating the physico-chemical similarity matrix (see S1 Text). Further, the perceptual and the physico-chemical clusters were compared using the Hubert index [49] (see Table 5). As it can be observed that, we have achieved a fairly high value of Hubert index. To state it clearly, the index calculates, if two molecules are in the same cluster in perceptual side, do they occupy same cluster in the physico-chemical side too and if they are not, are they in the different clusters in both the sides. A 0 indicates the least overlap and 1 the highest. The high values indicate that the molecules projected in the non-linear space from the perceptual and the physico-chemical side seem to occupy similar positions in a different projected space, although the number of clusters is different in both the cases. It suggests that the biological mechanism by which a signal is translated from physical to perceptual space is universal and structure-preserving (homomorphism). Homomorphism can lead us to develop statistical prediction mechanisms by which we can predict the perceptual qualities of a molecule by using its physico-chemical properties. To the best of our knowledge, it has not been observed for as many as 526 perceptual descriptors and large number of molecules earlier. It should be noted that, smell has acquired and inherited components, and therefore without considering the neural space, it cannot be perfectly predicted. In this sense, we also concentrate on the hard-wired part of olfactory perception which has been reported in literature [28]. We do not claim to suggest without any evidence from our side that pleasantness axis is hardwired as suggested in the seminal works by Khan *et al*.[28], Dielenberg and McGregor [50].

**Table 5:** The number of clusters obtained and the corresponding Hubert Index of the clusters.

Database	#Clusters Perceptual descriptors	#Clusters Structural	Hubert Index(HI)
**Flavornet**	12	9	0.518
**LJ**	17	17	0.750
**GoodScents**	17	17	0.718
**Sigma-Aldrich**	15	17	0.723
**SuperScent**	12	17	0.656
**Complete database**	17	17	0.733

### Predicting the perceptual qualities

We developed a machine learning framework analogous to the olfactory information processing for the assignment of perceptual qualities to the molecules. There are in total 526 perceptual descriptors in the databases. Hence, the similar molecules in the perceptual space (projected in different geometrical dimension) were clustered together and assigned the same class. Clustering was performed according to methods described in the previous section, i.e. the molecules were clustered using spectral clustering and X-means with BIC values (as criteria for getting the statistically optimal number of clusters, see S1 Text). The co-occurrence value was used for calculating the perceptual similarity matrix. This step can be assumed to be similar to an abstraction of perceptual classification in human brain, wherein, an abstract sense of a smell first evolves and later is decorrelated into several other annotations depending upon the repertoire of language and expertise [51,52]. We classified the molecules using the physicochemical features by a random forest classifier. A 10 fold cross validation approach was used for the classification. Cross validation was done inside the loop to avoid overfitting. ROC values for all the databases are > 0.7, with the poorest performance on SuperScent. Highest performance was achieved on GoodScents (0.83), although the number of molecules and perceptual descriptors is the highest here. Classification was also performed by using the features selected by a greedy correlation based feature selection algorithm (see S1 Text). It should be noted that, the feature selection and classification were done inside the cross-validation loop, so that, we don't over fit the model being developed. The feature selection was performed with a motive to enhance the ROC values and reduce the number of predictors. Although, much difference in the ROC values has not been observed, the numbers of features have been reduced significantly. It is very interesting to observe that only 3 features are necessary to achieve ROC value of 0.62 for the LJ database and similarly, for SuperScent this number is slightly higher (9) to achieve ROC value of 0.69. For all the other databases, the number of features varies from 49 (Flavornet) to 87 (GoodScents). The importance of features and their significance in perceptual inference requires further investigations. Table 6 lists the ROC values obtained and number of features selected for all the datasets. The high values of ROC indicated that it is possible to use selective physicochemical features to predict the perceptual qualities. The selected feature for all the databases separately and combined has been provided in S2 Table.

**Table 6:** The classification rate and ROC values with and without feature selection.

Database	ROC without feature selection	ROC with feature selection	# Features
**Flavornet**	0.787	0.798	49
**LJ**	0.733	0.623	3
**GoodScents**	0.83	0.829	87
**Sigma-Aldrich**	0.781	0.787	50
**SuperScent**	0.722	0.688	9
**Complete database**	0.809	0.817	61

## Discussion

In this work, we have accumulated publicly available odour database consisting of several hundred perceptual descriptors and several thousand compounds, one of the first attempts to analyse odour spaces on such a scale. We have systematically analysed the database characteristics and have shown by using network theoretical approaches that the perceptual universe or the verbal descriptions of the molecules are sparse and follow a power law. We have systematically compared the odour network with corresponding semantic networks and analysed, if the positioning of the perceptual descriptors is only due to their semantic relatedness. It is for the first time that it has been mathematically analysed on many databases as well as their integration. We have also tried to find perceptual communities and defined perceptual classes resulting from it.

The graphical representation provides a framework in which the perceptual relationships and the importance of a perceptual descriptor can be identified, which can be useful in understanding the relationship between language and olfactory perception. For example, our study clearly reveals that English as a language is not capable enough of representing the olfactory perception [53] due to the presence of some perceptual descriptors which are hubs or leaders in the networks, implying, “if you can’t understand, at least speak this”. The use of very specific words are rare, but when used, they are mostly used in combination with the more obvious descriptors like “fruit”, “floral”, “meat”, “green”, “wood” etc. As discussed earlier, it could confirm the "theory of least action" which suggests people tend to speak those words which they think will convey the broadest of information on a given topic, which is true for expert or a layman [40,41]. So, in the event of smell reporting the subject would tend to speak first the broader meaning words and then if asked or pressed more she/he would tend to speak more specific related words. There can be such studies for other languages too. We have also shown that the positioning of perceptual descriptors in the odour network is neither by chance, nor due to the semantic relatedness only. We have put forward objective similarity measures as well as a detailed look into the semantic networks. Clearly, the olfactory verbal descriptions are not driven by semantics. As far as we know, this has never been attempted before. Although, the scope of scaling such an experiment to a massive data domain from the olfactory as well as semantic database perspective still remains. Our study in this sense is also unique and notable as we have provided a framework which can act as the basis of theoretical understanding of perceptual descriptors and olfaction. The new perceptual descriptors can also be categorized or given a grouping with such kind of categorization. We have shown that perceptual descriptors form part of a hierarchical representation with some major descriptors dominating the perceptual space, hence indicating the possibility of properly defining perceptual classes and smaller specific sub-classes. The arguments presented in the work are strengthened by some earlier sensory works, which claim that the high-dimensional inputs from the sensory neurons are subsequently transformed by neural circuits. Friedrich *et al*. [54] have suggested that this transformation takes place in a way that the initial coarse odour representation refines over time and becomes increasingly odour-specific. A temporally hierarchical mechanism has been observed for the segmentation of odour and its identity information by Stopfer *et al*. [51]. This process also allows the system to extract olfactory features at several degrees of resolution [52]. While we don't tread into the concept of dimensionality of the perceptual descriptor space, of which there has been some considerable work most notably as described in the introduction section, by Castro *et al*. [11] and Zarzo *et al*.[10], who use NMF and PCA respectively and identify linear basis vectors which can account for the variability of perceptual descriptor data, some interpretation issues of these analyses is presented. First, the use of PCA, as aptly suggested by Castro *et al*. [11], since PCA and other factor analyses techniques do not constrain the original variables, they tend to occupy positions in smoothly distributed subspace. This tends to make interpretation of influence of the categorical variables difficult. While this analysis has proved very useful in overall interpretation of PC1 as "pleasantness" axis, the interpretation of higher dimensions PC3, PC4 is very difficult. The use of NMF although avoids this constraint but, the linear combination of variable obscures the positioning of individual descriptors and while mathematically it can be very sound, every basis vector has to be interpreted to some physical meaning. Our method avoids such kind of interpretation by positioning individual descriptors, eg: "lemony" close to "citrus" and both of them ultimately in "fruity" class indicating better representations. It should be noted that our method too is riddled with caveats, as the positioning of some descriptors like "fruity" and "meaty" etc. is bewildering. This has happened because, datasets themselves sometimes present some very ambiguous descriptors like putting "fruity" along side with "meat" etc. We have not attempted to remove such co-occurrences. The other issue for further study is better representation of co-occurrence values or derived metric. The large scale analysis of semantic descriptors and its comparison to the odour space can also be done.

The fact that, the molecules when projected in a non-linear space separately from the perceptual and physico-chemical side overlap significantly in a non-linear dimension, is significant. The high value of Hubert Index calculated affirms that, though the number of optimum clusters obtained for the databases are different, the molecules occupy similar clusters positions in different spaces. It affirms the structure preserving property of the odour spaces. This also gave us the encouragement to cluster molecules based on perceptual qualities and define categories to the molecules.

The research on finding a systematic relationship between physico-chemical properties of molecules and the perceptual descriptors [8,23,29,30,55] significantly contributed to our understanding of odour spaces, yet the works have concentrated on smaller databases (e.g. by Khan *et al*.). They also have concentrated on mainly predicting pleasantness of the molecules [29]. Schmuker *et al*. [30] have predicted perceptual qualities based on the Sigma-Aldrich database, but their main focus was on designing a virtual receptor and demonstrate its significance. Besides defining perceptual classes and its analysis, we have directly taken the large set of physico-chemical descriptors, subjected it to min-max normalization (scale between 0 and 1), and used them for classifying molecules based on their perceptual classes. We have obtained ROC value of 0.78 on Sigma-Aldrich database. The ROC value is also very good (0.8) for the combined database. Our work in this regards is significant from two perspectives. First, we accumulate and curate a diverse open domain dataset along with its physico-chemical properties. Second, we show that the molecules if projected to separate non-linear space in perceptual and physico-chemical dimensions, respectively occupy similar positions, hence a model can be developed which can predict the perceptual qualities of the molecules just by using the physico-chemical properties. This, we have validated by designing a random forest classifier and found very good ROC values for predicting perceptual qualities. We have successfully extracted some useful features for all the databases separately and together. Using this framework we can directly predict the perceptual qualities of a novel molecule using its physico-chemical feature. The selection of features has also thrown some interesting results i.e. maximum 87 features are needed to classify the compounds and for LJ only 3 features are responsible to get 0.6 ROC.

The clustering of molecules based on perceptual qualities can also act as a method for annotation of molecules and provide a base for designing odours and engineer smells. The comprehensive database along with the perceptual and physico-chemical properties can also act as a research base for the researchers and perfumers. The methods presented in this work could suggest and encourage new vistas in understanding language and perception.

Given this, it should be noted that an analysis is as good as the data it is subjected to. We have attempted to make sense of the uncurated large olfactory databases available on web. Moreover, the results need further investigation in terms of the correlations with neural signals and physico-chemical features. In this work, we have tried to minimize the errors that can creep into by not making any assumptions about the data and using robust statistical techniques.

## Methods

### Database description

For the present study, we have accumulated 5 different databases available publically viz. Leon and Johnson [19] (LJ), GoodScents [20], SuperScent [56], Flavornet [17] and Sigma-Aldrich [16]. We extracted the molecules and perceptual notations from these databases by both semi-automatic and manual methods. All these databases contain odoriferous molecules described by some words or perceptual descriptors apart from their molecular references such as CAS, SMILES, PMID and molecular weights. We wrote python and MATLAB scripts to extract the databases from html files from Flavornet, GoodScents, SuperScent and LJ. Data was extracted manually from Sigma-Aldrich by writing down the molecules and perceptual information. The pages at http://www.thegoodscentscompany.com contain references to the many subgroups of CAS numbers which contain lists of compounds with links to their descriptions ordered by CAS numbers. These cover all kinds of substances, from natural substances and extractives to pharmaceuticals. The links are http://www.thegoodscentscompany.com/allproc-1.html, http://www.thegoodscentscompany.com/allproc-2.html, and so on.

From these compounds, the CAS numbers were obtained and from each description page the textual information under "odor description" in the section "Organoleptic Properties" was downloaded. We wrote scripts to scrape all the pages which sometimes did not even contain any "odor descriptions". For example, http://www.thegoodscentscompany.com/data/rw1247381.html, is about a substance, CAS number 50-00-0, formaldehyde. It does not contain an odor information, therefore it was not included in the study, however, compound 50-21-5, 2-hydroxypropanoic acid, included an odour information (http://www.thegoodscentscompany.com/data/rw1007391.html). We converted the descriptions to lowercase and removed fill words and did some more semi-automatic curations as described in the next paragraph. For some compounds, it was impossible to obtain unique CAS, PMID or smiles notation so we removed them from our database. These molecular references were further used to extract freely available 1666 physico-chemical properties of the molecules using the E-DRAGON [57]. The properties with constant and missing values were removed to obtain 1489 physico-chemical feature sets. The descriptors were further normalized to be in the range of 0–1 (min-max normalizaton).

A user can search for the CAS number and obtain perceptual and physico-chemical description at http://odornetwork.com/index.jsp?page=aroma.

The web site may contain more compounds than analyzed here, because we have been adding them continuously.

It is imperative to note that the accuracy or variance of perceptual assignments in these databases cannot be ascertained due to the unavailability of information related to the psychophysical experiments in these databases. We removed some discrepancies in the molecular reference entries by comparing the given molecular references and the molecular weights calculated by E-DRAGON. Further, a series of semi-automatic methods were applied on these databases for creation of the perceptual feature sets. Firstly, the words describing the perceptual qualities of the molecules were tokenized to result in a set of perceptual descriptors (eg: for 2-methyl 2-pentenal perceptual qualities were specified as “powerful green grass somewhat fruity gassy” these were tokenized as “powerful” “green” “grass” “somewhat” “fruity” “gassy”). From these tokens, conjunctions (e.g. “and”), adverbs (e.g. “less”, “somewhat”), suffixes (e.g. “like”, “note”), auxiliary verbs (e.g. “has”) and some other words which don’t convey qualitative olfactory information (e.g. “over”, “preserves”, “powerful” and “other”) were further removed to get the feature vectors (eg: in this case it is “green” “grass” “fruity” “gassy”). It should be noted here that removing the adverbs may put us in danger of losing some valuable information about the perceptual description but adverbs like "somewhat" and "less" has come only once in the data set. While "like" has come 92 times, it only affirms a particular note rather creating confusion over it, the word "note" has come only 3 times across the databases. Further, the semantically equivalent perceptual descriptors such as “alcohol/alcoholic”, “fruit/fruity” etc. were merged. At last, the perceptual descriptors “odorless” and the associated molecules were removed. Further, the perceptual profile of a molecule was created by assigning a value of 1 to the descriptors that were used to describe the odour character of that molecule and 0 otherwise. Such kind of a representation yields *dichotomous matrices* for each database. We will refer these matrices as perceptual matrix (***A***) in the above text, where rows represent molecules (*m*) and columns percepts (*p*). Further, in order to capture the diversity of perceptual representation of each molecule, the perceptual descriptors were combined into a unified representation of matrix of 3016 molecules and 526 perceptual descriptors.

### Statistical analysis of perceptual data

The average number of percepts per molecule (*AP*_*m*_), average occurrence of a percept (*AM*_*p*_) and percentage of sparseness (*S*_*p*_) are defined as follows

$$AP_{m}=\frac{\sum_{i=1}^{m}\sum_{j=1}^{p}A_{i,j}}{m}$$ (1)

$$AM_{p}=\frac{\sum_{i=1}^{m}\sum_{j=1}^{p}A_{i,j}}{p}$$ (2)

$$Sp=\left(1-\;\frac{\;\;\sum_{i=1}^{m}\sum_{j=1}^{p}A_{i,j}}{m*p}\right)*100$$ (3)

Perceptual Co-occurrence network is calculated as C = A^T^ *A

### Network Characteristics of the perceptual network

The network parameters i.e. average degree, network diameter, average path length, graph density, clustering coefficient and assortativity are given in S1 Text.

For constructing a random network, Erdos Renyi G(n,m) model is used and it is described in S1 Text.

The Kolmogorov-Smirnov Test has been employed to test the degree distribution of network (S1 Text)

### Comparison with Semantic Networks

In order to compare the odour network with semantic network we have used the brown corpus and employed bag of words technique. The brown corpus is provided freely with Python NLTK suite. It consists of 500 samples of English language texts, totalling roughly 1 million words. This database has been used as a benchmark, and a bench work for many natural language processing tasks like Part of Speech Tagging, Semantic Analysis, word disambiguation etc. At first we identified the olfactory perceptual descriptors and searched the corpus for these descriptors. Obviously, the number of words matching, differed for different olfactory databases. Now, we used a window of 2,3,4 (these window sizes were chosen because the average number of perceptual descriptor for a molecule differed for different databases) to create 6 bag of words representations or associations. The words in the window were also searched for finding the match from olfactory perceptual descriptors. We further created a co-occurence network of these words i.e. how many times the olfactory perceptual descriptors came together in the corpus. This data was further analysed separately and compared with individual olfactory database and combined. The network parameters have been calculated for these networks similar as in Table 2 for which the description is available in the Supporting Information. It should be noted that in order to compare the semantic network and odour network, we kept the number and type of nodes same in both the cases. For an objective comparison of the corresponding networks we calculated a graph similarity measure, eigen similarity. Eigen similarity gives an unbounded measure in [0, ∞). Mathematically, if A_1_ And A_2_ are two adjacency matrices of the graphs which we are comparing, let L_1_ = D_1_- A_1_ and L_2_ = D_2_—A_2_ be laplacian of the graphs with D_1_ and D_2_ being corresponding diagonal matrices of the graphs. We define eigen value of laplacians and define similarity between graphs as

$$sim=\;\sum_{i}^{k}{\left(\lambda_{1i}-\;\lambda_{2i}\right)}^{2}$$ (4)

where k is the number of eigen vectors and it is chosen such that

 jmin{∑i=1kλji∑i=1nλji>0.9} (5)

for j = 1, 2 (corresponding to the two graphs that is being compared), we keep the top k eigen values that contain 90% of energy [58]. The values close to 0 indicate the graphs are very similar.

### Additional methods

For community detection, modularity maximization algorithm proposed by Blondel *et al*. [46] has been used (S1 Text). The resolution parameter was set to 1. The details of spectral clustering, correlation based feature selection, random forest classifier are also available in S1 Text.

## Supporting Information

S1 Table Top ten perceptual descriptors. This table shows the top ten occurring perceptual descriptors by frequency for all databases.
(DOCX)

S2 Table Features Selected using a greedy correlation based feature selection algorithm. This table shows the features selected for all the databases separately and complete database
(DOCX)

S3 Table Community information of the odour networks, with the perceptual descriptors according to the figure labels. (XLSX)

S4 Table The olfactory perceptual descriptors found in the brown database according to the database and window size of 2,3,4 as described in the main text. (XLSX)

S1 Text Supporting Information for Perceptual Network provides the additional details concerning the formation of network and the calculation of the network properties. The text also details the community detection algorithm and method undertaken to find power law. It also provides details concerning the spectral clustering and the novel method of combining spectral information with X-means clustering algorithm. It also describes the random forest classifier and correlation based feature selection algorithm. (DOCX)
