# Supplementary material for: Understanding the Odour Spaces: A Step towards Solving Olfactory Stimulus-Percept Problem
Source: PLoS One. 2015 Oct 20;10(10):e0141263. doi: 10.1371/journal.pone.0141263 (PMC4615634; doi:10.1371/journal.pone.0141263)
Supplement: S1 Table — This table shows the top ten occurring perceptual descriptors by frequency for all databases. (DOCX) [file pone.0141263.s001.docx]

**S1 Table. Top ten percepts.** This table shows the top ten occurring percepts by frequency for all databases

| **Flavornet** | **LJ** | **GoodScents** | **Sigma-Aldrich** | **SuperScent** | **Complete data** |
| --- | --- | --- | --- | --- | --- |
| Fruit | Sweet | Fruit | Sweet | Fruit | Fruit |
| Sweet | Fruit | Green | Fruit | Floral | Floral |
| Green | Green | Floral | Green | Balsam | Green |
| Fat | Floral | Sweet | Floral | Fat | Sweet |
| Floral | Pungent | Wood | Herb | Nut | Herb |
| Herb | Herb | Herb | Vegetable | Citrus | Wood |
| Spice | Fat | Fat | Apple | Herb | Fat |
| Wood | Wood | Sulfur | Meat | Chemical | Spice |
| Sulphur | Ether | Spice | Wood | Earth | Citrus |
| Pungent | Aromatic | Wax | Ethereal | Vegetable | Wax |
