# Supplementary material for: Understanding the Odour Spaces: A Step towards Solving Olfactory Stimulus-Percept Problem
Source: PLoS One. 2015 Oct 20;10(10):e0141263. doi: 10.1371/journal.pone.0141263 (PMC4615634; doi:10.1371/journal.pone.0141263)
Supplement: S2 Table — This table shows the features selected for all the databases separately and complete database (DOCX) [file pone.0141263.s002.docx]

**S2.1 Table. Flavornet.** This table shows the features selected from Flavornet database

| **Flavournet** | | |
| --- | --- | --- |
| 1 | Mp | Mean atomic polarizability (scaled on Carbon atom) Constitutional indices |
| 2 | nS | Number of Sulfur atoms Constitutional indices |
| 3 | nR05 | Number of 5-membered rings Ring descriptors |
| 4 | SNar | Narumi simple topological index (log function) Topological indices |
| 5 | HNar | Narumi harmonic topological index Topological indices |
| 6 | GNar | Narumi geometric topological index Topological indices |
| 7 | SPI | Superpendentic index Topological indices |
| 8 | TPC | Total path count Walk and path counts |
| 9 | X1MulPer | Multiplicative perturbation connectivity index Connectivity indices |
| 10 | IC5 | Information Content index (neighborhood symmetry of 5-order) Information indices |
| 11 | SIC5 | Structural Information Content index (neighborhood symmetry of 5-order) Information indices |
| 12 | CIC0 | Complementary Information Content index (neighborhood symmetry of 0-order) Information indices |
| 13 | VR3_D | Logarithmic Randic-like eigenvector-based index from topological distance matrix 2D matrix-based descriptors |
| 14 | SpMax_L | Leading eigenvalue from Laplace matrix 2D matrix-based descriptors |
| 15 | SM5_L | Spectral moment of order 5 from Laplace matrix 2D matrix-based descriptors |
| 16 | AVS_X | Average vertex sum from chi matrix 2D matrix-based descriptors |
| 17 | Chi_H2 | Randic-like index from reciprocal squared distance matrix 2D matrix-based descriptors |
| 18 | SM5_Dt | Spectral moment of order 5 from detour matrix 2D matrix-based descriptors |
| 19 | EE_Dz(Z) | Estrada-like index (log function) from Barysz matrix weighted by atomic number 2D matrix-based descriptors |
| 20 | Ho_Dz(m) | Hosoya-like index (log function) from Barysz matrix weighted by mass 2D matrix-based descriptors |
| 21 | SpDiam_Dz(e) | Spectral diameter from Barysz matrix weighted by Sanderson electronegativity 2D matrix-based descriptors |
| 22 | SpAD_Dz(e) | Spectral absolute deviation from Barysz matrix weighted by Sanderson electronegativity 2D matrix-based descriptors |
| 23 | SpMAD_Dz(e) | Spectral mean absolute deviation from Barysz matrix weighted by Sanderson electronegativity 2D matrix-based descriptors |
| 24 | AVS_Dz(p) | Average vertex sum from Barysz matrix weighted by polarizability 2D matrix-based descriptors |
| 25 | VR1_B(m) | Randic-like eigenvector-based index from Burden matrix weighted by mass 2D matrix-based descriptors |
| 26 | HyWi_B(v) | Hyper-Wiener-like index (log function) from Burden matrix weighted by van der Waals volume 2D matrix-based descriptors |
| 27 | SpPos_B(v) | Spectral positive sum from Burden matrix weighted by van der Waals volume 2D matrix-based descriptors |
| 28 | SpPosA_B(v) | Normalized spectral positive sum from Burden matrix weighted by van der Waals volume 2D matrix-based descriptors |
| 29 | SM3_B(v) | Spectral moment of order 3 from Burden matrix weighted by van der Waals volume 2D matrix-based descriptors |
| 30 | AVS_B(p) | Average vertex sum from Burden matrix weighted by polarizability 2D matrix-based descriptors |
| 31 | SM5_B(p) | Spectral moment of order 5 from Burden matrix weighted by polarizability 2D matrix-based descriptors |
| 32 | J_B(i) | Balaban-like index from Burden matrix weighted by ionization potential 2D matrix-based descriptors |
| 33 | ATSC3p | Centred Broto-Moreau autocorrelation of lag 3 weighted by polarizability 2D autocorrelations |
| 34 | MATS8p | Moran autocorrelation of lag 8 weighted by polarizability 2D autocorrelations |
| 35 | MATS3i | Moran autocorrelation of lag 3 weighted by ionization potential 2D autocorrelations |
| 36 | GATS6e | Geary autocorrelation of lag 6 weighted by Sanderson electronegativity 2D autocorrelations |
| 37 | GGI9 | Topological charge index of order 9 2D autocorrelations |
| 38 | SpMax3_Bh(p) | Largest eigenvalue n. 3 of burden matrix weighted by polarizability burden eigenvalues |
| 39 | SpMin1_Bh(v) | Smallest eigenvalue n. 1 of burden matrix weighted by van der waals volume burden eigenvalues |
| 40 | SpMin7_Bh(e) | Smallest eigenvalue n. 7 of burden matrix weighted by sanderson electronegativity burden eigenvalues |
| 41 | P_VSA_i_2 | P_VSA-like on ionization potential |
| 42 | P_VSA_s_6 | P_VSA-like on I-state |
| 43 | SpMax_AEA(ed) | Leading eigenvalue from augmented edge adjacency mat. Weighted by edge degree edge adjacency indices |
| 44 | SpMaxA_AEA(ed) | Normalized leading eigenvalue from augmented edge adjacency mat. Weighted by edge degree edge adjacency indices |
| 45 | Chi0_EA(bo) | Connectivity-like index of order 0 from edge adjacency mat. Weighted by bond order edge adjacency indices |
| 46 | SM14_EA(ed) | Spectral moment of order 14 from edge adjacency mat. Weighted by edge degree edge adjacency indices |
| 47 | SM04_EA(dm) | Spectral moment of order 4 from edge adjacency mat. Weighted by dipole moment edge adjacency indices |
| 48 | Eig03_AEA(ri) | Eigenvalue n. 3 from augmented edge adjacency mat. Weighted by resonance integral edge adjacency indices |
| 49 | SpPos_G/D | Spectral positive sum from distance/distance matrix 3D matrix-based descriptors |

**S2.2Table. LJ.** This table shows the features selected from LJ database

| **LJ** | | |
| --- | --- | --- |
| 1 | ZM1 | First Zagreb index Topological indices |
| 2 | Xt | Total structure connectivity index Topological indices |
| 3 | SM07_AEA(dm) | Spectral moment of order 7 from augmented edge adjacency mat. Weighted by dipole moment edge adjacency indices |

**S2.3Table. GoodScents.** This table shows the features selected from GoodScents database

| **GoodScents** | | |
| --- | --- | --- |
| 1 | AMW | Average molecular weight Constitutional indices |
| 2 | nAB | Number of aromatic bonds Constitutional indices |
| 3 | nBR | Number of Bromine atoms Constitutional indices |
| 4 | nR12 | Number of 12-membered rings Ring descriptors |
| 5 | ZM1 | First Zagreb index Topological indices |
| 6 | Qindex | Quadratic index Topological indices |
| 7 | GNar | Narumi geometric topological index Topological indices |
| 8 | SPI | Superpendentic index Topological indices |
| 9 | CSI | Eccentric connectivity index Topological indices |
| 10 | X3v | Valence connectivity index of order 3 Connectivity indices |
| 11 | AAC | Mean information index on atomic composition Information indices |
| 12 | IC5 | Information Content index (neighborhood symmetry of 5-order) Information indices |
| 13 | QW_L | Quasi-Wiener index (Kirchhoff number) from Laplace matrix 2D matrix-based descriptors |
| 14 | SpPos_L | Spectral positive sum from Laplace matrix 2D matrix-based descriptors |
| 15 | SpDiam_L | Spectral diameter from Laplace matrix 2D matrix-based descriptors |
| 16 | H_X | Harary-like index from chi matrix 2D matrix-based descriptors |
| 17 | Chi_X | Randic-like index from chi matrix 2D matrix-based descriptors |
| 18 | EE_X | Estrada-like index (log function) from chi matrix 2D matrix-based descriptors |
| 19 | J_H2 | Balaban-like index from reciprocal squared distance matrix 2D matrix-based descriptors |
| 20 | Ho_Dt | Hosoya-like index (log function) from detour matrix 2D matrix-based descriptors |
| 21 | SpAD_D/Dt | Spectral absolute deviation from distance/detour matrix 2D matrix-based descriptors |
| 22 | AVS_Dz(Z) | Average vertex sum from Barysz matrix weighted by atomic number 2D matrix-based descriptors |
| 23 | SpMax_Dz(Z) | Leading eigenvalue from Barysz matrix weighted by atomic number 2D matrix-based descriptors |
| 24 | SpMAD_Dz(Z) | Spectral mean absolute deviation from Barysz matrix weighted by atomic number 2D matrix-based descriptors |
| 25 | SM3_Dz(Z) | Spectral moment of order 3 from Barysz matrix weighted by atomic number 2D matrix-based descriptors |
| 26 | WiA_Dz(m) | Average Wiener-like index from Barysz matrix weighted by mass 2D matrix-based descriptors |
| 27 | SpPos_Dz(m) | Spectral positive sum from Barysz matrix weighted by mass 2D matrix-based descriptors |
| 28 | Ho_Dz(m) | Hosoya-like index (log function) from Barysz matrix weighted by mass 2D matrix-based descriptors |
| 29 | AVS_Dz(v) | Average vertex sum from Barysz matrix weighted by van der Waals volume 2D matrix-based descriptors |
| 30 | SpPosA_Dz(v) | Normalized spectral positive sum from Barysz matrix weighted by van der Waals volume 2D matrix-based descriptors |
| 31 | VE2_Dz(v) | Average coefficient of the last eigenvector from Barysz matrix weighted by van der Waals volume 2D matrix-based descriptors |
| 32 | SpDiam_Dz(e) | Spectral diameter from Barysz matrix weighted by Sanderson electronegativity 2D matrix-based descriptors |
| 33 | SpMAD_Dz(e) | Spectral mean absolute deviation from Barysz matrix weighted by Sanderson electronegativity 2D matrix-based descriptors |
| 34 | Ho_Dz(e) | Hosoya-like index (log function) from Barysz matrix weighted by Sanderson electronegativity 2D matrix-based descriptors |
| 35 | EE_Dz(e) | Estrada-like index (log function) from Barysz matrix weighted by Sanderson electronegativity 2D matrix-based descriptors |
| 36 | SM5_Dz(e) | Spectral moment of order 5 |
| 37 | VR3_Dz(e) | Logarithmic Randic-like eigenvector-based index from Barysz matrix weighted by Sanderson electronegativity 2D matrix-based desc... |
| 38 | Wi_Dz(p) | Wiener-like index from Barysz matrix weighted by polarizability 2D matrix-based descriptors |
| 39 | WiA_Dz(p) | Average Wiener-like index from Barysz matrix weighted by polarizability 2D matrix-based descriptors |
| 40 | AVS_Dz(p) | Average vertex sum from Barysz matrix weighted by polarizability 2D matrix-based descriptors |
| 41 | H_Dz(p) | Harary-like index from Barysz matrix weighted by polarizability 2D matrix-based descriptors |
| 42 | VE3_B(m) | Logarithmic coefficient sum of the last eigenvector from Burden matrix weighted by mass 2D matrix-based descriptors |
| 43 | Wi_B(v) | Wiener-like index from Burden matrix weighted by van der Waals volume 2D matrix-based descriptors |
| 44 | SpAbs_B(v) | Graph energy from Burden matrix weighted by van der Waals volume 2D matrix-based descriptors |
| 45 | AVS_B(p) | Average vertex sum from Burden matrix weighted by polarizability 2D matrix-based descriptors |
| 46 | J_B(i) | Balaban-like index from Burden matrix weighted by ionization potential 2D matrix-based descriptors |
| 47 | Chi_B(s) | Randic-like index from Burden matrix weighted by I-State 2D matrix-based descriptors |
| 48 | ATS4i | Broto-Moreau autocorrelation of lag 4 (log function) weighted by ionization potential 2D autocorrelations |
| 49 | ATSC2e | Centred Broto-Moreau autocorrelation of lag 2 weighted by Sanderson electronegativity 2D autocorrelations |
| 50 | ATSC8s | Centred Broto-Moreau autocorrelation of lag 8 weighted by I-state 2D autocorrelations |
| 51 | MATS3m | Moran autocorrelation of lag 3 weighted by mass 2D autocorrelations |
| 52 | MATS4m | Moran autocorrelation of lag 4 weighted by mass 2D autocorrelations |
| 53 | MATS6m | Moran autocorrelation of lag 6 weighted by mass 2D autocorrelations |
| 54 | MATS7m | Moran autocorrelation of lag 7 weighted by mass 2D autocorrelations |
| 55 | MATS6e | Moran autocorrelation of lag 6 weighted by Sanderson electronegativity 2D autocorrelations |
| 56 | MATS1p | Moran autocorrelation of lag 1 weighted by polarizability 2D autocorrelations |
| 57 | MATS4i | Moran autocorrelation of lag 4 weighted by ionization potential 2D autocorrelations |
| 58 | MATS1s | Moran autocorrelation of lag 1 weighted by I-state 2D autocorrelations |
| 59 | GATS6e | Geary autocorrelation of lag 6 weighted by Sanderson electronegativity 2D autocorrelations |
| 60 | GATS4p | Geary autocorrelation of lag 4 weighted by polarizability 2D autocorrelations |
| 61 | GATS4s | Geary autocorrelation of lag 4 weighted by I-state 2D autocorrelations |
| 62 | GGI2 | Topological charge index of order 2 2D autocorrelations |
| 63 | GGI3 | Topological charge index of order 3 2D autocorrelations |
| 64 | GGI4 | Topological charge index of order 4 2D autocorrelations |
| 65 | SpMax3_Bh(p) | Largest eigenvalue n. 3 of burden matrix weighted by polarizability burden eigenvalues |
| 66 | SpMax3_Bh(s) | Largest eigenvalue n. 3 of burden matrix weighted by i-state burden eigenvalues |
| 67 | SpMin4_Bh(v) | Smallest eigenvalue n. 4 of burden matrix weighted by van der waals volume burden eigenvalues |
| 68 | SpMin3_Bh(e) | Smallest eigenvalue n. 3 of burden matrix weighted by sanderson electronegativity burden eigenvalues |
| 69 | SpMin8_Bh(s) | Smallest eigenvalue n. 8 of burden matrix weighted by i-state burden eigenvalues |
| 70 | SpMaxA_AEA(ed) | Normalized leading eigenvalue from augmented edge adjacency mat. Weighted by edge degree edge adjacency indices |
| 71 | SpMaxA_AEA(dm) | Normalized leading eigenvalue from augmented edge adjacency mat. Weighted by dipole moment edge adjacency indices |
| 72 | SpMAD_AEA(dm) | Spectral mean absolute deviation from augmented edge adjacency mat. Weighted by dipole moment edge adjacency indices |
| 73 | SpMax_AEA(ri) | Leading eigenvalue from augmented edge adjacency mat. Weighted by resonance integral edge adjacency indices |
| 74 | Chi0_EA(ri) | Connectivity-like index of order 0 from edge adjacency mat. Weighted by resonance integral edge adjacency indices |
| 75 | SM04_EA(dm) | Spectral moment of order 4 from edge adjacency mat. Weighted by dipole moment edge adjacency indices |
| 76 | SM06_EA(ri) | Spectral moment of order 6 from edge adjacency mat. Weighted by resonance integral edge adjacency indices |
| 77 | SM13_EA(ri) | Spectral moment of order 13 from edge adjacency mat. Weighted by resonance integral edge adjacency indices |
| 78 | SM02_AEA(ed) | Spectral moment of order 2 from augmented edge adjacency mat. Weighted by edge degree edge adjacency indices |
| 79 | QZZm | Quadrupole z-component value / weighted by mass Geometrical descriptors |
| 80 | QZZe | Quadrupole z-component value / weighted by Sanderson electronegativity Geometrical descriptors |
| 81 | ChiA_G | Average Randic-like index from geometrical matrix 3D matrix-based descriptors |
| 82 | J_RG | Balaban-like index from reciprocal squared geometrical matrix 3D matrix-based descriptors |
| 83 | HyWi_RG | Hyper-Wiener-like index from reciprocal squared geometrical matrix 3D matrix-based descriptors |
| 84 | J_G/D | Balaban-like index from distance/distance matrix 3D matrix-based descriptors |
| 85 | SpPos_G/D | Spectral positive sum from distance/distance matrix 3D matrix-based descriptors |
| 86 | SpPosLog_G/D | Logarithmic spectral positive sum from distance/distance matrix 3D matrix-based descriptors |
| 87 | SpMax_G/D | Leading eigenvalue from distance/distance matrix 3D matrix-based descriptors |

**S2.4Table. Sigma-Aldrich.** This table shows the features selected from Sigma-Aldrich database

| **Sigma-Aldrich** | | |
| --- | --- | --- |
| 1 | nBR | Number of Bromine atoms Constitutional indices |
| 2 | HNar | Narumi harmonic topological index Topological indices |
| 3 | LPRS | Log of product of row sums (PRS) Topological indices |
| 4 | X2v | Valence connectivity index of order 2 Connectivity indices |
| 5 | X3v | Valence connectivity index of order 3 Connectivity indices |
| 6 | IDE | Mean information content on the distance equality Information indices |
| 7 | QW_L | Quasi-Wiener index (Kirchhoff number) from Laplace matrix 2D matrix-based descriptors |
| 8 | SM3_L | Spectral moment of order 3 from Laplace matrix 2D matrix-based descriptors |
| 9 | VR2_L | Normalized Randic-like eigenvector-based index from Laplace matrix 2D matrix-based descriptors |
| 10 | VR3_L | Logarithmic Randic-like eigenvector-based index from Laplace matrix 2D matrix-based descriptors |
| 11 | SpAD_H2 | Spectral absolute deviation from reciprocal squared distance matrix 2D matrix-based descriptors |
| 12 | SpMAD_Dz(Z) | Spectral mean absolute deviation from Barysz matrix weighted by atomic number 2D matrix-based descriptors |
| 13 | SpPos_Dz(m) | Spectral positive sum from Barysz matrix weighted by mass 2D matrix-based descriptors |
| 14 | VE1_Dz(m) | Coefficient sum of the last eigenvector from Barysz matrix weighted by mass 2D matrix-based descriptors |
| 15 | AVS_Dz(v) | Average vertex sum from Barysz matrix weighted by van der Waals volume 2D matrix-based descriptors |
| 16 | VE2_Dz(v) | Average coefficient of the last eigenvector from Barysz matrix weighted by van der Waals volume 2D matrix-based descriptors |
| 17 | SpMaxA_Dz(e) | Normalized leading eigenvalue from Barysz matrix weighted by Sanderson electronegativity 2D matrix-based descriptors |
| 18 | SpMAD_Dz(e) | Spectral mean absolute deviation from Barysz matrix weighted by Sanderson electronegativity 2D matrix-based descriptors |
| 19 | Ho_Dz(e) | Hosoya-like index (log function) from Barysz matrix weighted by Sanderson electronegativity 2D matrix-based descriptors |
| 20 | VR3_Dz(e) | Logarithmic Randic-like eigenvector-based index from Barysz matrix weighted by Sanderson electronegativity 2D matrix-based desc... |
| 21 | AVS_B(v) | Average vertex sum from Burden matrix weighted by van der Waals volume 2D matrix-based descriptors |
| 22 | SpAD_B(i) | Spectral absolute deviation from Burden matrix weighted by ionization potential 2D matrix-based descriptors |
| 23 | J_B(s) | Balaban-like index from Burden matrix weighted by I-State 2D matrix-based descriptors |
| 24 | ATSC5i | Centred Broto-Moreau autocorrelation of lag 5 weighted by ionization potential 2D autocorrelations |
| 25 | MATS3m | Moran autocorrelation of lag 3 weighted by mass 2D autocorrelations |
| 26 | MATS4m | Moran autocorrelation of lag 4 weighted by mass 2D autocorrelations |
| 27 | MATS6m | Moran autocorrelation of lag 6 weighted by mass 2D autocorrelations |
| 28 | MATS6e | Moran autocorrelation of lag 6 weighted by Sanderson electronegativity 2D autocorrelations |
| 29 | MATS7i | Moran autocorrelation of lag 7 weighted by ionization potential 2D autocorrelations |
| 30 | GATS2e | Geary autocorrelation of lag 2 weighted by Sanderson electronegativity 2D autocorrelations |
| 31 | GATS8e | Geary autocorrelation of lag 8 weighted by Sanderson electronegativity 2D autocorrelations |
| 32 | GATS4i | Geary autocorrelation of lag 4 weighted by ionization potential 2D autocorrelations |
| 33 | GATS4s | Geary autocorrelation of lag 4 weighted by I-state 2D autocorrelations |
| 34 | GGI2 | Topological charge index of order 2 2D autocorrelations |
| 35 | SpMax7_Bh(e) | Largest eigenvalue n. 7 of burden matrix weighted by sanderson electronegativity burden eigenvalues |
| 36 | SpMax6_Bh(i) | Largest eigenvalue n. 6 of burden matrix weighted by ionization potential burden eigenvalues |
| 37 | SpMaxA_AEA(ed) | Normalized leading eigenvalue from augmented edge adjacency mat. Weighted by edge degree edge adjacency indices |
| 38 | SpDiam_AEA(dm) | Spectral diameter from augmented edge adjacency mat. Weighted by dipole moment edge adjacency indices |
| 39 | Chi0_EA | Connectivity-like index of order 0 from edge adjacency mat. Edge adjacency indices |
| 40 | Chi0_EA(ri) | Connectivity-like index of order 0 from edge adjacency mat. Weighted by resonance integral edge adjacency indices |
| 41 | SM02_EA(ed) | Spectral moment of order 2 from edge adjacency mat. Weighted by edge degree edge adjacency indices |
| 42 | SM06_EA(bo) | Spectral moment of order 6 from edge adjacency mat. Weighted by bond order edge adjacency indices |
| 43 | SM15_EA(ri) | Spectral moment of order 15 from edge adjacency mat. Weighted by resonance integral edge adjacency indices |
| 44 | SM02_AEA(ed) | Spectral moment of order 2 from augmented edge adjacency mat. Weighted by edge degree edge adjacency indices |
| 45 | SM03_AEA(ed) | Spectral moment of order 3 from augmented edge adjacency mat. Weighted by edge degree edge adjacency indices |
| 46 | SM10_AEA(ed) | Spectral moment of order 10 from augmented edge adjacency mat. Weighted by edge degree edge adjacency indices |
| 47 | Eig05_AEA(dm) | Eigenvalue n. 5 from augmented edge adjacency mat. Weighted by dipole moment edge adjacency indices |
| 48 | DISPp | Displacement value / weighted by polarizability Geometrical descriptors |
| 49 | J_RG | Balaban-like index from reciprocal squared geometrical matrix 3D matrix-based descriptors |
| 50 | SpAbs_G/D | Graph energy from distance/distance matrix 3D matrix-based descriptors |

**S2.5 Table. SuperScent.** This table shows the features selected from SuperScent database

| **SuperScent** | |  |
| --- | --- | --- |
| 1 | HNar | Narumi harmonic topological index Topological indices |
| 2 | QW_L | Quasi-Wiener index (Kirchhoff number) from Laplace matrix 2D matrix-based descriptors |
| 3 | SM4_X | Spectral moment of order 4 from chi matrix 2D matrix-based descriptors |
| 4 | VE3_B(m) | Logarithmic coefficient sum of the last eigenvector from Burden matrix weighted by mass 2D matrix-based descriptors |
| 5 | SpDiam_EA(bo) | Spectral diameter from edge adjacency mat. Weighted by bond order edge adjacency indices |
| 6 | SM07_EA(bo) | Spectral moment of order 7 from edge adjacency mat. Weighted by bond order edge adjacency indices |
| 7 | SM10_EA(ri) | Spectral moment of order 10 from edge adjacency mat. Weighted by resonance integral edge adjacency indices |
| 8 | SM02_AEA(ed) | Spectral moment of order 2 from augmented edge adjacency mat. Weighted by edge degree edge adjacency indices |
| 9 | DISPp | Displacement value / weighted by polarizability Geometrical descriptors |

**S2.6Table. Complete.** This table shows the features selected from Complete e database

| **Complete** | | |
| --- | --- | --- |
| 1 | nAB | Number of aromatic bonds Constitutional indices |
| 2 | nBR | Number of Bromine atoms Constitutional indices |
| 3 | nR12 | Number of 12-membered rings Ring descriptors |
| 4 | ZM1 | First Zagreb index Topological indices |
| 5 | ZM2 | Second Zagreb index Topological indices |
| 6 | HNar | Narumi harmonic topological index Topological indices |
| 7 | SPI | Superpendentic index Topological indices |
| 8 | PJI2 | 2D Petitjean shape index Topological indices |
| 9 | CSI | Eccentric connectivity index Topological indices |
| 10 | X3v | Valence connectivity index of order 3 Connectivity indices |
| 11 | AAC | Mean information index on atomic composition Information indices |
| 12 | TIC1 | Total Information Content index (neighborhood symmetry of 1-order) Information indices |
| 13 | STN_L | Spanning tree number (log function) from Laplace matrix 2D matrix-based descriptors |
| 14 | SpDiam_L | Spectral diameter from Laplace matrix 2D matrix-based descriptors |
| 15 | VR2_L | Normalized Randic-like eigenvector-based index from Laplace matrix 2D matrix-based descriptors |
| 16 | AVS_X | Average vertex sum from chi matrix 2D matrix-based descriptors |
| 17 | EE_X | Estrada-like index (log function) from chi matrix 2D matrix-based descriptors |
| 18 | Wi_Dz(Z) | Wiener-like index from Barysz matrix weighted by atomic number 2D matrix-based descriptors |
| 19 | AVS_Dz(Z) | Average vertex sum from Barysz matrix weighted by atomic number 2D matrix-based descriptors |
| 20 | SpMax_Dz(Z) | Leading eigenvalue from Barysz matrix weighted by atomic number 2D matrix-based descriptors |
| 21 | WiA_Dz(m) | Average Wiener-like index from Barysz matrix weighted by mass 2D matrix-based descriptors |
| 22 | SpPos_Dz(m) | Spectral positive sum from Barysz matrix weighted by mass 2D matrix-based descriptors |
| 23 | VE1_Dz(m) | Coefficient sum of the last eigenvector from Barysz matrix weighted by mass 2D matrix-based descriptors |
| 24 | AVS_Dz(v) | Average vertex sum from Barysz matrix weighted by van der Waals volume 2D matrix-based descriptors |
| 25 | SpPosA_Dz(v) | Normalized spectral positive sum from Barysz matrix weighted by van der Waals volume 2D matrix-based descriptors |
| 26 | SpDiam_Dz(e) | Spectral diameter from Barysz matrix weighted by Sanderson electronegativity 2D matrix-based descriptors |
| 27 | EE_Dz(e) | Estrada-like index (log function) from Barysz matrix weighted by Sanderson electronegativity 2D matrix-based descriptors |
| 28 | WiA_Dz(p) | Average Wiener-like index from Barysz matrix weighted by polarizability 2D matrix-based descriptors |
| 29 | AVS_Dz(p) | Average vertex sum from Barysz matrix weighted by polarizability 2D matrix-based descriptors |
| 30 | H_Dz(p) | Harary-like index from Barysz matrix weighted by polarizability 2D matrix-based descriptors |
| 31 | VE3_B(m) | Logarithmic coefficient sum of the last eigenvector from Burden matrix weighted by mass 2D matrix-based descriptors |
| 32 | WiA_B(p) | Average Wiener-like index from Burden matrix weighted by polarizability 2D matrix-based descriptors |
| 33 | AVS_B(p) | Average vertex sum from Burden matrix weighted by polarizability 2D matrix-based descriptors |
| 34 | J_B(p) | Balaban-like index from Burden matrix weighted by polarizability 2D matrix-based descriptors |
| 35 | ATS8m | Broto-Moreau autocorrelation of lag 8 (log function) weighted by mass 2D autocorrelations |
| 36 | ATSC8s | Centred Broto-Moreau autocorrelation of lag 8 weighted by I-state 2D autocorrelations |
| 37 | MATS3m | Moran autocorrelation of lag 3 weighted by mass 2D autocorrelations |
| 38 | MATS4m | Moran autocorrelation of lag 4 weighted by mass 2D autocorrelations |
| 39 | MATS6m | Moran autocorrelation of lag 6 weighted by mass 2D autocorrelations |
| 40 | MATS1v | Moran autocorrelation of lag 1 weighted by van der Waals volume 2D autocorrelations |
| 41 | MATS6e | Moran autocorrelation of lag 6 weighted by Sanderson electronegativity 2D autocorrelations |
| 42 | MATS3i | Moran autocorrelation of lag 3 weighted by ionization potential 2D autocorrelations |
| 43 | MATS4i | Moran autocorrelation of lag 4 weighted by ionization potential 2D autocorrelations |
| 44 | GATS2e | Geary autocorrelation of lag 2 weighted by Sanderson electronegativity 2D autocorrelations |
| 45 | GATS6e | Geary autocorrelation of lag 6 weighted by Sanderson electronegativity 2D autocorrelations |
| 46 | GATS4p | Geary autocorrelation of lag 4 weighted by polarizability 2D autocorrelations |
| 47 | GATS4s | Geary autocorrelation of lag 4 weighted by I-state 2D autocorrelations |
| 48 | GGI4 | Topological charge index of order 4 2D autocorrelations |
| 49 | SpMax3_Bh(p) | Largest eigenvalue n. 3 of burden matrix weighted by polarizability burden eigenvalues |
| 50 | P_VSA_p_4 | P_VSA-like on polarizability |
| 51 | Chi0_EA | Connectivity-like index of order 0 from edge adjacency mat. Edge adjacency indices |
| 52 | Chi0_EA(ri) | Connectivity-like index of order 0 from edge adjacency mat. Weighted by resonance integral edge adjacency indices |
| 53 | SM03_EA(dm) | Spectral moment of order 3 from edge adjacency mat. Weighted by dipole moment edge adjacency indices |
| 54 | SM14_EA(ri) | Spectral moment of order 14 from edge adjacency mat. Weighted by resonance integral edge adjacency indices |
| 55 | SM02_AEA(ed) | Spectral moment of order 2 from augmented edge adjacency mat. Weighted by edge degree edge adjacency indices |
| 56 | QZZm | Quadrupole z-component value / weighted by mass Geometrical descriptors |
| 57 | ChiA_G | Average Randic-like index from geometrical matrix 3D matrix-based descriptors |
| 58 | J_RG | Balaban-like index from reciprocal squared geometrical matrix 3D matrix-based descriptors |
| 59 | J_G/D | Balaban-like index from distance/distance matrix 3D matrix-based descriptors |
| 60 | SpPos_G/D | Spectral positive sum from distance/distance matrix 3D matrix-based descriptors |
| 61 | SpPosA_G/D | Normalized spectral positive sum from distance/distance matrix 3D matrix-based descriptors |
