# Supplementary material for: Understanding the Odour Spaces: A Step towards Solving Olfactory Stimulus-Percept Problem
Source: PLoS One. 2015 Oct 20;10(10):e0141263. doi: 10.1371/journal.pone.0141263 (PMC4615634; doi:10.1371/journal.pone.0141263)
Supplement: S1 Text — (DOCX) [file pone.0141263.s005.docx]

## Supporting Information

Ritesh Kumar^*^, Benjamin Auffarth, Rishemjit Kaur, Amol P. Bhondekar

E-mail: riteshkr@csio.res.in

**S1 Perceptual Network**

There can be different techniques in order to further understand a dichotomous matrix (A matrix of 1’s and 0’s). A very famous method is using Principal Component Analysis (PCA) [1]. It basically uses a matrix factorization technique and assumes the data to be normally distributed. The other popular techniques are using Latent Semantic Analysis (LSA) [2] and the very effective Latent Dritchlet Allocation (LDA) [3]. Each of these techniques uses different forms of matrix factorization after first pre-processing the data. The most difficult part in using these methods is the interpretation as the data represented after transformations are linear combinations of original feature vectors. Hence, we sought to design a network based approach to understand perceptual organisation. The molecule-percept matrix extracted from the different databases forms a bipartite network. The bipartite networks are the networks formed between two different types of nodes; popular examples include collaboration networks of scientists (edge between scientists and publications) [4], actors (actors and movies) [5] and disease network[6]. We transformed the molecule-percept bipartite network into a weighted perceptual network in which the weight W_ij_ is the number of molecules shared between two percepts. So, if a bipartite network is denoted by matrix A then the perceptual co-occurrence network is found by equation S1 as:

C = A* A^T^ (S1)

**S2 Calculation of network properties**

We have calculated different network properties of the perceptual network

**Average degree (A)** is defined by equation S2 as follows:

A = $\frac{2*|E|}{|V|}$ (S2)

Where, E is the number of edges and V is the number of nodes in an undirected graph. We included the influence of weights for the network by multiplying A by connection strength defined as mean weight of connections per node on the graph.

**Network diameter (N_d_)** is the longest of all the calculated shortest path of nodes in the network. It is defined by equation S3

N_d_ = ${max}_{i,j}I(i,j)$ (S3)

Here, I(I,j ) denotes the length of shortest path between nodes *i* and *j*.

**Average path length (N_l_)** is the average distance between any two nodes in the network.

N_l_ $= \frac{\sum_{i\geq j} I(i,j)}{\frac{n(n-1)}{2}}$ (S4)

I(I,j ) denotes the length of shortest path between nodes i and j. The denominator in the equation S10 is the total possible number of edges in the network.

**Graph density (D_g_)** for a simple undirected graph is defined as in equation S5, where, E is the number of edges and V, the number of nodes.

D_g_ = $\frac{2*|E|}{|V|(\left| V \right|-1)}$ (S5)

**Clustering coefficient** quantifies the extent to which the neighbours of the concerned node are connected to each other as in equation S6.

cl*_i_* (g) = $\frac{number of triangles connected to vertex i}{number of triples centered at i}$ (S6)

The average clustering coefficient can be defined according to equation S7.

${cl}^{avg}$ = 1/n$\sum_{i} {cl}_{i}(g)$ (S7)

Where cl_i_(g) is the clustering coefficient of a node i.

The clustering coefficient can be used to differentiate the properties of the nodes from a random network. We have calculated the weighted clustering coefficient proposed by Barrat *et al.*[7].

**Assortativity** or often called as assortative mixing is the property of the nodes of a network to preferentially attach to nodes which are similar in some way. The assortative coefficient can be defined as the Pearson correlation coefficient of degree between pairs of linked nodes; it may be defined as in equation S8.

$r = \frac{\sum_{jk} jk(e_{jk}-q_{j}q_{k})}{\sigma_{q}^{2}}$ (S8)

Where, $q_{k}$ is the distribution of remaining degree i.e. it concerns the number of edges leaving the node other than the one that connects the pair such that $q_{k}$ = $\frac{p_{k+1}}{\sum_{j\geq1} p_{j}}$ . $e_{jk}$ is the joint probability distribution of the remaining degrees of the two vertices such that it sums to 1.

**S3 Comparison with null model**

For constructing a random network we have used a null model using Erdos Renyi G(n,m) framework. In the G(n,m) model a graph is chosen uniformly from all possible collection of graphs which have n nodes and m edges which means the probability of an edge is equal in the graph.

1000 instances of G(n,m) models were created and the clustering coefficients of all the models were calculated and then a comparison was done using z-test.

**S4 Fitting the degree distribution**

The degree distributions of the networks were fit according to the power law. In mathematics a quantity follows a power law if it is sampled from a probability distribution such that

P(x) = k *x^-α^ (S9)

Where k is the proportionality constant and α is the exponent or scaling parameter of the distribution. The scaling parameters generally lie between 2 and 3. There are networks in the real world which exhibit exponents to be close to 2. In order to test how well the power law distribution fits our data we performed Kolmogorov Smirnov (KS)[8,9] test to check if the observed data and the one generated from power law come from the same distribution. We also cross checked this result with other alternative distributions viz. exponential, exponential with X_min_, lognormal and lognormal with X_min_. These distributions were chosen because of their similarity to the power law distribution. We performed KS tests to determine whether the observed data and the synthetic data sets generated from the other distributions with our chosen parameters are similar. The points which obtained significant values were only considered as measurement of good fit. The one with significantly higher number of points having passed the test is considered to be proper fit of the data. Table S1 shows the number of times significant values have been obtained and the best fit distribution for all the dataset.

**Table S1: The bestfit distribution along with the number of times out of 2500 P>0.10**

| **Dataset** | **TP*** | **exponential** | **exponential with X_min_** | **lognormal** | **lognormal with X_min_** |
| --- | --- | --- | --- | --- | --- |
| Flavornet | **2485** | 0 | 443 | 76 | 0 |
| GoodScents | **2115** | 0 | 240 | 268 | 274 |
| LJ | **2475** | 0 | 705 | 0 | 0 |
| Sigma-Aldrich | **2464** | 550 | 9 | 951 | 0 |
| SuperScent | 2338 | 348 | 1700 | **2447** | 2446 |
| Complete Database | **2128** | 0 | 27 | 67 | 0 |

**TP* = Truncated Power Law**

**S5 Community detection**

The study of community detection in the networks involves partitioning the graph into communities based on some objective function where intra-community connections are dense and inter-community connections are very sparse. Modularity can be defined as the measurement of strength of a network into modules. It compares the density of intra community links to inter community links. It is often used as an objective function to maximise for detecting community structure. It can be defined as the fraction of edges that fall within the given groups subtracted by the expected fraction of a null model. The objective function can be put as in equation S10.

$Q= \frac{1}{2m}\sum_{i,j} \left[ A_{ij}- \frac{k_{i}k_{j}}{2m} \right]\delta\left( c_{i},c_{j} \right)$ (S10)

Where A_ij_ represents the weight of edge between nodes *i* and *j*, $k_{i}= \sum_{j} A_{ij}$is the weighted degree of node i, $m= \frac{1}{2}\sum_{ij} A_{ij}$ , *c_i_* is the community to which node i is assigned and $\delta\left( c_{i},c_{j} \right)$ =1 if $c_{i}=c_{j}$ and 0 otherwise.

The modularity maximization algorithm used in this work is proposed by Blondel *et al.*[10]*.* It consists of two phases. In first phase, initially all nodes are assigned to separate communities and further the nodes are merged into same community if there is a modularity gain in doing so. This process is continued for all the nodes till no further gain in modularity is achieved. i.e. local maxima of modularity is achieved. In second phase, a new network is build where the communities found in the first phase act as nodes of this new network. Then again, the first phase is applied to this new network and this process is repeated till no change in modularity is observed. This algorithm is fast and also, its intrinsic multi-level nature helps in resolving the so-called resolution limit problem.

**S6 Spectral Clustering**

Spectral clustering has become very popular among researchers in the recent years. It clusters data points based on their connectivity not necessarily their compactness. This algorithm has following steps.

1. For n data points, build the affinity/similarity matrix. For the physicochemical data, affinity matrix was build based on the Euclidean distance between data points and then locally scaled using method proposed by Manor *et al.*[11]. The affinity *A_ij_* between a pair of data points is calculated as follows:

$A_{ij}=exp(\frac{-d^{2} (s_{i}{, s}_{j})}{\sigma_{i}\sigma_{j}})$ (S11)

Where for each data point *s_i_*, the local scaling parameter σ_i_ is calculated on the basis of local statistics of its neighbourhood.

For perceptual data, the affinity between two data points (molecules) is defined as the number of precepts shared between them.

1. The normalized Laplacian affinity matrix L is constructed, L = D^-1/2^A D^-1/2^ , where D is a diagonal matrix with D_ii_ = $\sum_{j=1}^{n} A_{ij}$
2. Compute eigenvectors of L
3. Select *K* largest eigenvectors and renormalize the rows of selected eigenvectors to form matrix *Y*.
4. Treat each row of *Y* as a point and cluster using x-means.

The clustering was performed on both sides (i.e. perceptual and physico-chemical) for all the databases. The number of eigenvectors which maximizes the similarity between clusters thus obtained for all the datasets was chosen as the optimum value. The Huberts index[12] as defined in equation S12 was used for measuring the similarity of clusters and value of k equal to 10 was chosen.

HI = ((a+d) - (b+c))/ (a + b+ c + d), 0 ≤HI≤1 (S12)

Considering two partitions C1, C2

a: number of points belonging to same cluster in C1 and C2 both

b: number of points belonging to same cluster in C1 but different in C2

c: number of points belonging to different clusters in C1 but same in C2

d: number of points belonging to different clusters in C1 and different in C2

HI close to 1 indicates high agreement between two clusters partitions.

**S6.1 X-means**

X-means algorithm proposed by Pelleg*et al* [13]*.*is used for clustering purpose. It is an extended k-means where the no of clusters are estimated using BIC. Initially it sets the no. of clusters equal to the minimum no of clusters, and then applies k-means to it. Further it is followed by splitting process based on the BIC. It sequentially keeps on splitting the clusters till it reaches the maximum number of clusters. It embeds the dataset into a multi-resolutional kd-tree and stores sufficient statistics at its nodes which helps in improving the speed of k-means.

The minimum no. of clusters, maximum no of clusters, maximum leaf size, minimum box width, cut off factor, maximum iterations and number of splits were set to 1, 40, 40, 0.03, 0.5, 200 and 6 respectively.

**S7 Feature selection and classification**

We classified the molecules using the physicochemical features. We performed a simple correlation based feature selection algorithm in conjunction with random forest classifier using 10 fold cross validation approach. We repeated the procedure without doing feature selection too.

**S7.1 Correlation based feature selection**

This algorithm evaluates the worth of a subset of attributes by considering the individual predictive ability of each feature along with the degree of redundancy between them. Subsets of features that are highly correlated with the class while having low inter-correlation are preferred [14,15]. The algorithm iteratively adds features with the highest correlation with the class as long as there is already no feature having higher correlation with the feature in question. The features are selected by greedy hill-climbing with backtracking. We have set 5 as the number of consecutive non-improving nodes allowed, which controls the level of backtracking done. The algorithm starts with the empty set of attributes and searches in the forward direction. We have used WEKA machine learning platform for this task[14].

**S7.2 Random Forest**

Random forest is one of the class of ensemble learning in which a large number of weak learners, here decision trees are constructed[16]. The random forest algorithm at first creates subsets of data with replacement and then creates a random set of features from these data or a set of decision trees. The decision of class is done by voting mechanism. The maximum number of trees constructed here is 200. We have used WEKA machine learning platform for this task[14].

**References**

1. Jolliffe I. Principal component analysis. Wiley Online Library; 2005.

2. Landauer TK, Foltz PW, Laham D. An introduction to latent semantic analysis. Discourse Process. Taylor & Francis; 1998;25: 259–284.

3. Blei DM, Ng AY, Jordan MI. Latent dirichlet allocation. J Mach Learn Res. JMLR. org; 2003;3: 993–1022.

4. Newman MEJ. Scientific collaboration networks. I. Network construction and fundamental results. Phys Rev E. APS; 2001;64: 16131.

5. Barabâsi A-L, Jeong H, Néda Z, Ravasz E, Schubert A, Vicsek T. Evolution of the social network of scientific collaborations. Phys A Stat Mech its Appl. Elsevier; 2002;311: 590–614.

6. Goh K-I, Cusick ME, Valle D, Childs B, Vidal M, Barabási A-L. The human disease network. Proc Natl Acad Sci. National Acad Sciences; 2007;104: 8685–8690.

7. Barrat A, Barthelemy M, Pastor-Satorras R, Vespignani A. The architecture of complex weighted networks. 2003; 1–7. doi:10.1073/pnas.0400087101

8. Clauset A, Shalizi CR, Newman MEJ. Power-law distributions in empirical data. SIAM Rev. SIAM; 2009;51: 661–703.

9. Goldstein ML, Morris SA, Yen GG. Problems with fitting to the power-law distribution. Eur Phys J B-Condensed Matter Complex Syst. Springer; 2004;41: 255–258.

10. Blondel VD, Guillaume J-L, Lambiotte R, Lefebvre E. Fast unfolding of communities in large networks. J Stat Mech Theory Exp. 2008;2008: P10008. doi:10.1088/1742-5468/2008/10/P10008

11. Zelnik-Manor L, Perona P. Self-tuning spectral clustering. Advances in neural information processing systems. 2004. pp. 1601–1608.

12. Dubes RC. How many clusters are best?-an experiment. Pattern Recognit. Elsevier; 1987;20: 645–663.

13. Pelleg D, Moore AW. X-means: Extending K-means with Efficient Estimation of the Number of Clusters. ICML. 2000. pp. 727–734.

14. Hall M, Frank E, Holmes G, Pfahringer B, Reutemann P, Witten IH. The WEKA data mining software: an update. ACM SIGKDD Explor Newsl. ACM; 2009;11: 10–18.

15. Hall MA, Smith LA. Feature Subset Selection : A Correlation Based Filter Approach. : 1–4.

16. Breiman L. Random forests. Mach Learn. Springer; 2001;45: 5–32.
